# Comparative Study of the Cytokine/Chemokine Response in Children with Differing Disease Severity in Enterovirus 71-Induced Hand, Foot, and Mouth Disease

**DOI:** 10.1371/journal.pone.0067430

**Published:** 2013-06-28

**Authors:** Yan Zhang, Haiying Liu, Linghang Wang, Fan Yang, Yongfeng Hu, Xianwen Ren, Guojun Li, Yang Yu, Shaoxia Sun, Yufen Li, Xinchun Chen, Xingwang Li, Qi Jin

**Affiliations:** 1 MOH Key Laboratory of Systems Biology of Pathogens, Institute of Pathogen Biology, Chinese Academy of Medical Sciences & Peking Union Medical College, Beijing, China; 2 Infectious Diseases Centre, Beijing Ditan Hospital, Beijing, China; 3 Paediatric Department, LinYi People’s Hospital, Lin Yi, Shan Dong Province, China; 4 Shenzhen-Hong Kong Institute of Infectious Disease, Shenzhen Third People’s Hospital, Guangdong Medical College, Shenzhen, China; University of Rochester, United States of America

## Abstract

**Background:**

Enterovirus 71 (EV71) infection can lead to a rapidly progressing, life-threatening, and severe neurological disease in young children, including the development of human hand, foot, and mouth disease (HFMD). This study aims to further characterize the specific immunological features in EV71–mediated HFMD patients presenting with differing degrees of disease severity.

**Methodology:**

Comprehensive cytokine and chemokine expression were broadly evaluated by cytokine antibody array in EV71–infected patients hospitalized for HFMD compared to Coxsackievirus A16-infected patients and age-matched healthy controls. More detailed analysis using Luminex-based cytokine bead array was performed in EV71–infected patients stratified into diverse clinic outcomes. Additionally, immune cell frequencies in peripheral blood and EV71–specific antibodies in plasma were also examined.

**Principal Findings:**

Expression of several cytokines and chemokines were significantly increased in plasma from EV71–infected patients compared to healthy controls, which further indicated that: (1) GM-CSF, MIP-1β, IL-2, IL-33, and IL-23 secretion was elevated in patients who rapidly developed disease and presented with uncomplicated neurological damage; (2) G-CSF and MCP-1 were distinguishably secreted in EV71 infected very severe patients presenting with acute respiratory failure; (3) IP-10, MCP-1, IL-6, IL-8, and G-CSF levels were much higher in cerebrospinal fluid than in plasma from patients with neurological damage; (4) FACS analysis revealed that the frequency of CD19^+^HLADR^+^ mature B cells dynamically changed over time during the course of hospitalization and was accompanied by dramatically increased EV71–specific antibodies. Our data provide a panoramic view of specific immune mediator and cellular immune responses of HFMD and may provide useful immunological profiles for monitoring the progress of EV71–induced fatal neurological symptoms with acute respiratory failure.

## Introduction

Among the various enterovirus serotypes that can cause human hand, foot, and mouth disease (HFMD), the picornaviridae family member Enterovirus 71 (EV71) is particularly associated with this disease. Indeed, EV71 was found to be the underlying cause of several large HFMD outbreaks since it was first identified in California in 1969 [1]–[4]. As a neurotropic enterovirus, EV71 infection is unpredictable, causing diverse clinical progression and outcomes ranging from mild to fatal, and can also lead to long-term neurological sequelae [5]–[8].

China has recently experienced several HFMD epidemics in which a significant number of fatalities occurred among young children. Among them, nearly 90% were positive for the human EV strains, EV71 and Coxsackievirus A16 (CA16) [9]. Although most HFMD cases are mild, the disease can potentially be life threatening. This is especially true in infants and children <5 years old, as they are particularly susceptible to viral infection. HFMD may prove fatal in this age group within 2–3 days following the onset of severe neurological complications, such as brainstem encephalitis (BE) [10], acute respiratory failure and/or fatal pulmonary edema (PE) [10], and hemorrhage [5], [7], [11], [12]. Understanding the elusive mechanisms underlying host immune responses to EV71 infection, including identifying the important immune mediators and the status of cell-mediated immunity, will be essential to design effective vaccines and antiviral therapies.

Previous studies indicate that cellular immunity is indispensable for controlling the development and severity of EV71–mediated disease [11], [13], [14]. Cytokines and chemokines––important immune mediators that can be used to predict infection and tissue damage [15]––are implicated in both the systemic and central nervous system (CNS) inflammation that accompanies EV71 infection. Several independent studies showed that EV71–infected patients with BE and PE exhibit various elevated chemokines and cytokines, including interferon (IFN)-γ (a Th1 cytokine), IL-6 (a pleiotropic cytokine), IL-1β (a pro-inflammatory cytokine), IL-10 (an immunoregulatory cytokine), IL-13 (a Th2 cytokine), and the chemokines IL-8 and IP-10 [11], [13], [14], among others. While these independent studies provided some illustrative examples of altered immune mediators, their methods varied among the reports. A larger, more comprehensive study allowing within-study evaluation of immune mediators would be useful to understand the distinct clinical characteristics of EV71-infected patients.

We thereby recruited 153 HFMD patients upon hospitalization and 19 healthy controls within this single study. Among the HFMD patients, sequencing analysis confirmed that 99 were EV71–positive, 31 were CA16–positive and 23 were other enterovirus-positive. We initially screened a broad range of 120 immune factors in a few representative patients by performing cytokine antibody array on patient and healthy control plasma. Following, a panel of 30 cytokines/chemokines was selected and further evaluated by Luminex-based cytokine bead array analysis in a larger cohort of 69 HFMD patients as compared to age-matched healthy controls with no history of HFMD. Furthermore, alterations in cytokine/chemokine levels were also found among patients with diverse clinical characteristics. In particular, GM-CSF, MIP-1β, IL-2, IL-33, IL-23, G-CSF, and MCP-1 secretion were enhanced in HFMD disease presenting with increased neurological damage as compared to controls. Moreover, dynamics of cytokine and chemokine expression during the period of hospitalization was also examined in EV71 infected HFMD patients. These data not only provide a panoramic view of cytokine/chemokine responses elicited by EV71 infection and the associated neurologic sequelae in HFMD but also contribute to an in-depth understanding of the cellular immunity generated in response to HFMD.

## Materials and Methods

### Patient Grouping

A total of 153 children were clinically diagnosed with HFMD and grouped according to their clinical characteristics: mild (M) cases (fever; rash or vesicles on the hands and/or feet; or aphthous ulcers on the tongue and oral mucosa, n = 86), severe (S) cases (fever, with or without rash and uncomplicated neurological presentations, including myoclonus, vomiting, ataxia, irritability, and hypersomnia, n = 59), and very severe (VS) cases (quickly developed acute respiratory failure and PE; all patients selected in this group were admitted into the intensive care unit, n = 8). All patients were identified according to the Ministry of Health diagnostic criteria of HFMD (2008), china (http://www.moh.gov.cn/mohbgt/s9503/200812/38494.shtml). Among them, 99 EV71– and 31 CA16–positive patients were identified; samples were collected from each individual and studied. Patient characteristics are summarized in Table 1 and 2. Patient ages ranged from 5 months to 8 years old, and the average duration of treatment during hospitalization were 4 days for mild patients, 9 days for severe patients, and 18 days for very severe patients. Also, 19 age-matched healthy children (Con) with no history of HFMD were included as controls.

**Table 1:** Patient Characteristics of 153 recruited patients.

Group	Severity	M/F	NO	AOA*	ATD*
EV71+	VS	5/3	8	1.13±0.32	15∼20days
	S	20/16	36	2.36±1.66	7∼10days
	M	33/22	55	2.13±1.28	4∼5days
CA16+	S	6/5	11	2.80±1.82	7∼10days
	M	15/5	20	2.41±1.45	4∼5days
Others	–	12/11	23	2.32±1.62	–
Total No.			153		

* AOA = Average of age.

* ATD = Average of therapy duration.

One hundred and fifty-three clinically diagnosed HFMD patients were recruited and classified into 3 groups depending on the clinical severity of disease and degrees of neurological damage.

**Table 2:** Patient Characteristics for immunological examination.

Group	Severity	M/F	NO	AOA*	ATD*
EV71+	VS	5/3	8	1.13±0.32	15∼20days
	S	14/14	28	2.51±1.73	7∼10days
	M	13/10	23	2.04±1.32	4∼5days
CA16+	S	4/0	4	2.00±0	7∼10days
	M	3/3	6	2.50±0.84	4∼5days
H	–	10/9	19	7.61±4.94	–
Total No.			88		

* AOA = Average of age.

* ATD = Average of therapy duration.

Among 153 recruit patients, blood samples from 59 EV71-positive HFMD patients, 10 CA16-positive HFMD patients and 19 healthy controls were successfully obtained and used for immunological examination.

### Ethics Statement

This study was approved by and carried out under the guidelines of the Ethical Committee of the Beijing Ditan Hospital, Shandong LinYi People’s Hospital and the Shenzhen–Hong Kong Institute of Infectious Disease, Shenzhen Third People’s Hospital. Written informed consent was obtained from the next of kin on behalf of children participants involved in this study.

### Sample Collection

Clinical samples included throat swabs, rectal swabs, and blood from all patients; cerebrospinal fluid (CSF) was collected only from severe patients. For some mild cases, blood was collected upon admission and discharge from the hospital. For some severe patients, blood was collected at 4 time points, including admission, 24 h, 48–72 h, and discharge; CSF was obtained at the time of initial presentation. All blood samples were collected in Vacutainer-EDTA tubes (Becton Dickinson Medical Devices Co Ltd.). Whole blood was centrifuged at 2,000 rpm for 10 min at room temperature. Plasma was separated and stored in aliquots at −80°C until analysis. The remainder of the blood sample was resuspended in PBS buffer and carefully layered into a tube containing an equal volume of Ficoll buffer. Peripheral blood lymphocytes were isolated from the resulting Ficoll gradient in accordance with the manufacturer’s recommendations (Ficoll-Paque PLUS, GE healthcare 71-7167-00).

### RNA Extraction and Identifying Viral Etiology

Viral diagnosis was performed on at least one clinical specimen per individual (throat swab, stool, CSF, or plasma) by reverse transcriptase PCR (RT-PCR) and sequencing with specific transcript primers (F: 5′-GGAGATAGGGTRGCAGATGTAAT-3′; R: 5′-ATTTCCCAAGAGTAGTGATCGC-3′) designed to amplify EV71 viral mRNA. Semi-nested RT-PCR on the 5′ partial region of VP1 with sense (GCIATGYTIGGIACICAYRT; CCAGCACTGACAGCAGYNGARAYNGG) and antisense (CICCIGGIGGIAYRWACAT; TACTGGACCACCTGGNGGNAYRWACAT) primers was used to type EV directly from specimens as previously described [16]. Viral RNA was extracted from samples submitted for clinical diagnosis using Qiagen viral RNA kit (Qiagen, Inc.), and RT-PCR was performed using Qiagen OneStep RT-PCR Kit (Qiagen, Inc.) according to the manufacturer’s recommendations. PCR products were sequenced using Big Dye v3.0 sequencing kits and an ABI3730 automated sequencer (Applied Biosciences). Nucleotide sequences were aligned using MEGA4 sequence analysis software.

### Human Cytokine Antibody Array

Patient plasma was collected from 4 mild EV71–positive HFMD patients without neurological presentation, 5 severe EV71 positive HFMD patients with apparent neurological damage and symptoms, and 2 age-matched healthy controls. Their cytokine profiles were analyzed by a semi-quantitative human cytokine antibody array that detected 120 cytokines within one experiment (AAH-CYT-1000, Raybiotech Cytokine Antibody Array C series 1000). All sample measurements were performed according to the manufacturer’s specifications. Signals were normalized using internal, positive, and negative controls included on the array and analyzed using the Raybiotech analysis tool. Data was further analyzed using a data analysis program based on Microsoft Excel technology specifically designed to analyze Raybiotech Antibody Array C Series. Figures were designed by Matlab software.

### Multi-Cytokine/Chemokine Measurement by Luminex-based Cytokine Bead Array

All plasma and CSF samples from patients and controls were screened for cytokine and chemokine expression by using the Human Cytokine/Chemokine Lincoplex Kit (MPXHCYTO-60K-23, MPXHCYTO-60K-15 & MPXHCYP2-62K-3; Millipore, Linco Research Inc.). All cytokines and chemokines are classified in Table 3. Assays were performed according to the manufacturer’s recommendations. A standard curve covering the 3.2–4,000 pg/mL or 3.2–10,000 pg/mL concentration range was generated by serially diluting reconstituted standards. Plates were read by the Luminex 200 system (Luminex Corporation). Data were collected and analyzed by Luminex xPONENT software. A five-parameter regression formula was used to calculate sample concentration from the standard curves.

**Table 3:** Thirty cytokines/chemokines of various functional groups were analyzed by Luminex-based cytokine bead array.

Functional classification	Selected factors
ProInflammatory & Th cytokines	TNF-a, IL-1α,IL-1β, IFN-γ, IL-4,IL-23, IL-33, IL-12p40,IL-12p70, IL-2,IL-13,IL-5
AntiInflammatory cytokines	IL-10,IL-20
Other pleiotropic cytokines	G-GCF,GM-CSF,IFNα2α,IL-17,IL-6,IL-16,IL-7
Chemokines	IL-8,MCP-1,MIP-1a,MIP-1β,Eotaxin,Flt-3L, Fractalkine,IP-10,hBCA

### VP-1–specific Antibody ELISA Assay

Recombinant VP-1 protein derived from EV71 (kindly gifted by Prof. Xinchun Chen from Shenzhen Third People’s Hospital, China) was adjusted to 100 ng/mL (for detecting IgG) and 20 ng/mL (for detecting IgM) in coating buffer (15 mM Na_2_CO_3_, 30 mM NaHCO_3_, pH 9.6). Flat-bottomed 96-well Polysorp immunoplates were coated with the VP-1 antigen and incubated overnight at 4°C. Plates were washed with PBS-Tween20 and blocked with 200 µL/well of 1% BSA/PBS for 1 h at room temperature. After washing, 50 µL of 1∶50 diluted plasma was added into each well and incubated for 1 h at 37°C. Goat anti-human IgG antibody or goat anti-human IgM antibody (Beijing GBI-GBI Biotech Co., Ltd.) was added to the plates at 50 µL as a secondary antibody. Plates were incubated for 20 min at 37°C and washed. Signals were developed with 50 µL TMB (eBioscience, 00-4210-56) for 10 min at room temperature, and then 2M H_2_SO_4_ was added to stop the reaction. Absorbance of each well was read at 450 nm using an ELISA Microplate Reader (BIO-RAD, Model 680).

### Flow Cytometry Analysis

Peripheral blood lymphocytes from 13 severe patients, 10 mild patients, and 3 healthy controls were freshly separated by Ficoll, as described above, for flow cytometric analysis. Cells were resuspended in PBS and stained with the appropriate antibodies. The following fluorescently labeled mAbs were used: anti-CD4–PE, anti-CD4–FITC (BD, #555346, 555347), anti-CD8–APC (BD, 555346), anti-HLADR–FITC (BD, 555811), and anti-CD19–APC (eBioscience, 17-0199-73). Mouse IgM, IgG1, and IgG2a isotype antibody conjugates (BD) were included to determine background fluorescence. At least 0.1×10^6^ cells were acquired by the FACS CantoII instrument (BD Biosciences). Data was analyzed using FACSDiva software.

### Virus Load Determination

Total RNA was extracted from throat swab of HFMD patients using Qiagen RNeasy Mini kit (Qiagen, Inc.), and cDNA was synthesized using PrimeScript™ II 1st Strand cDNA Synthesis Kit (Takara). Real time quantitative RT-PCR was performed using Power SYBR Green PCR Master Mix (Applied Biosystems) according to the manufacturer’s recommendations. The following primers were used: forward, 5_-AGTATGATTGAGAC ACGCTG-3_ and reverse, 5_-GCAACAAAAGTGAACTCTGC-3_ to amplify the region of nt 2622–2854 in EV71 strain Shzh-98 genome sequence. The obtained segment was cloned into pGEM-T Easy vector to construct a plasmid as standard [17]. After amplification, the standard curve ranged from 4.8×10^8^ to 4.8×10^3^ copies as described in previous publication.

### Statistical Analysis

The non-parametric ANOVA test, Wilcoxon’s Sign Rank Test and unpaired t-test were used for statistic analysis. The differences between two groups were determined by non-parametric ANOVA test and t-test respectively and the differences between every two time points from individuals were determined by Wilcoxon’s Sign Rank Test. Figures were finished by GraphPad prism 5 (GraphPad software) and Matlab software. Data are presented as the mean ± SEM, and differences achieving values of P<0.05 were considered significant.

## Results

### EV71–mediated HFMD Induces a Broad Range of Cytokines and Chemokines

To initially identify the viral etiology underlying HFMD from each individual patient in our recruited study population, we performed RT-PCR and nucleic-acid sequencing on at least one clinical specimen (throat swab, stool, CSF, or plasma) [9]. All 8 patients classified as having very severe HFMD with respiratory failure (8% of the 99 EV71–positive HFMD cases) were included among the 99 HFMD patients found to be positive for EV71 (Table 1), suggesting that EV71 infection remains the major cause of clinically severe HFMD in China. While EV71 infected the majority of patients, EV71–negative HFMD patients were infected by a variety of other respiratory viruses or enterovirus serotypes (data not shown). We therefore focused on evaluating the cytokine and chemokine responses induced in EV71–infected HFMD patients.

**Figure 1:**
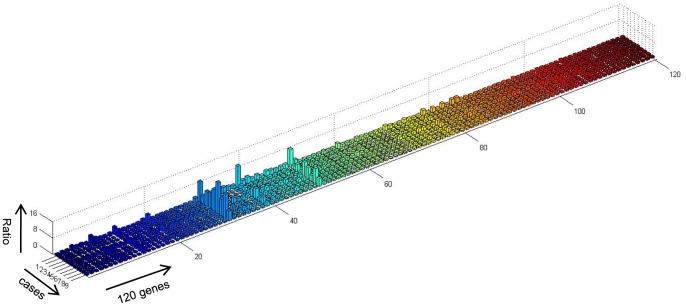
Cytokine antibody array broadly screened for changes in 120 immune mediators in EV71-positive HFMD patients. Cytokine antibody array screened 120 immune factors in 4 mild patients (no neurological syndromes), 5 severe patients (with neurological syndromes), and 2 healthy controls. Data are presented as ratios of the normalized signals for each factor in each of the nine individual patients to the mean concentration obtained from the healthy controls. Data were analyzed by EXCEL and Matlab software.

Cytokines and chemokines secreted during HFMD are key properties to evaluate because they can help us to understand disease development and inform ways to develop treatments and cures. We first performed a broad cytokine antibody array of 120 immune mediators on a single chip in order to generally understand the peripheral cytokine/chemokine immune responses in patients before treatment, including 4 mild patients and 5 severe patients with neurological manifestation 2–3 days after fever onset. The data in Figure 1 are presented as the ratio of the normalized signals of each factor from each patient as compared to the mean concentration from healthy controls. Thirteen factors were strikingly elevated in HFMD patients compared to healthy controls when using a value-cutoff ratio of ≥1.5; IL-2 (max = 15.47× higher than control) and GM-CSF (max = 8.75× higher than control) were particularly elevated. To further confirm which factors were significantly altered in a larger population of recruited patients, a panel of 30 cytokines and chemokines (including pro-inflammatory, Th, anti-inflammatory, and pleiotropic cytokines, Table 3) was selected and examined by Luminex-based cytokine bead array. Among them, 13 factors were expressed at low levels and were not significantly different from controls (data not shown). Some patient groups exhibited differences in the remaining 17 factors, and their plasma concentrations are detailed with their respective *p* values in Table S1.

**Figure 2:**
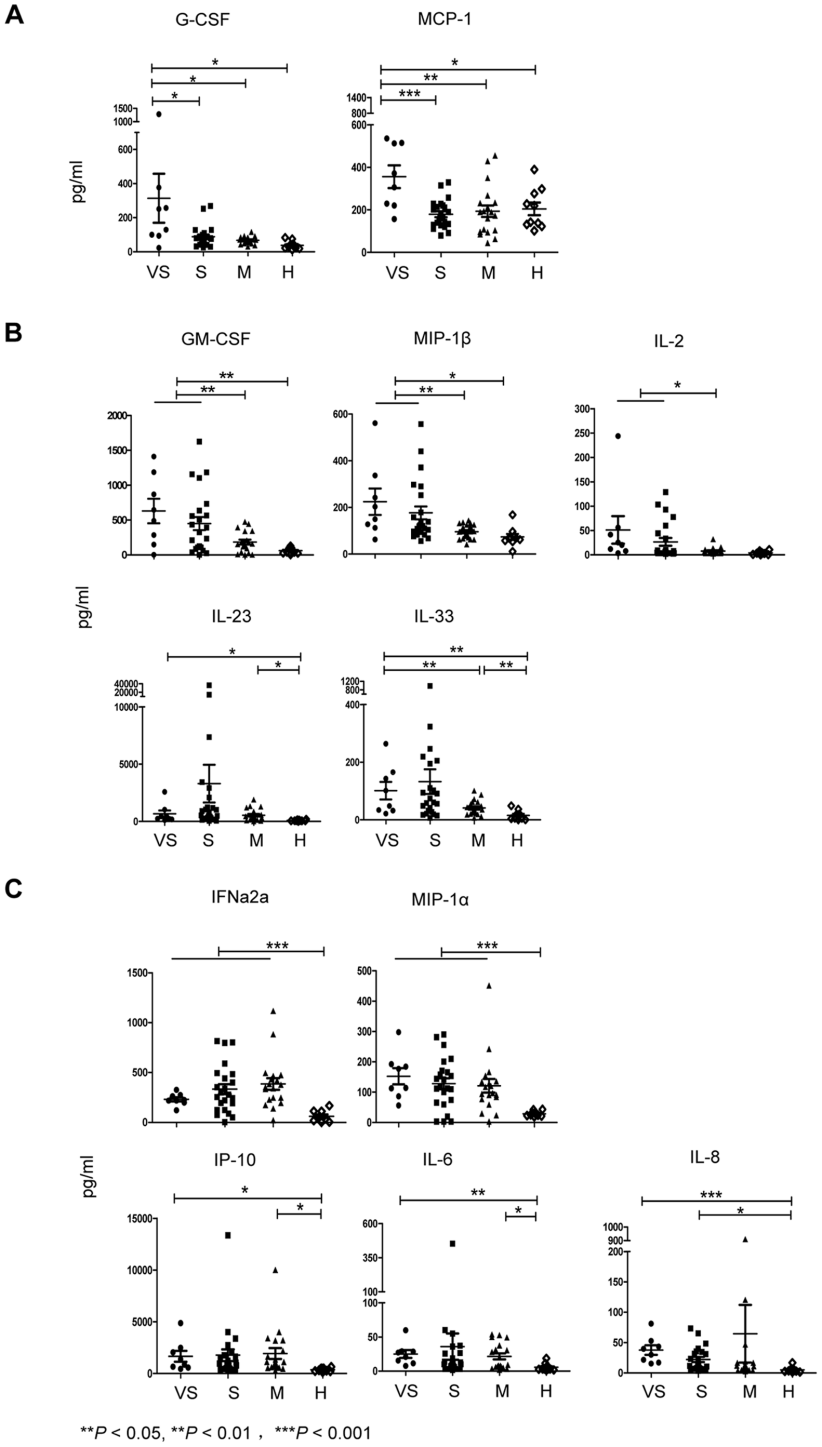
Peripheral cytokine/chemokine expression was compared between EV71-positive HFMD patient groups stratified by disease severity. (A, B, C) The profiles of 12 cytokines/chemokines were significantly elevated in plasma samples from recruited EV71-positive HFMD patients: VS = severe patients with pulmonary edema, n = 8; S = severe patients with uncomplicated neurological manifestations, n = 23; M = mild patients without neurological syndromes, n = 19; and H = healthy controls, n = 10. (A) G-CSF and MCP-1 were significantly increased in plasma from very severe patients with acute respiratory failure. (B) Cytokines/chemokines were markedly elevated in plasma of both very severe and severe patients. (C) Cytokines/chemokines were significantly enhanced in all HFMD patients compared to healthy controls. The unpaired Student’s *t*-test and non-parametric ANOVA test were used to compare variables between any two groups and *P* value from unpaired Student’s *t*-test analysis was presented in figures. *P<0.05, **P<0.01,***P<0.001. Each assay was performed duplicate and data are representative of at least 2 independent experiments.

Among the 17 factors found to change in HFMD patients, we first noted a marked increase in G-CSF (mean VS:S concentration ratio = 3.57) and MCP-1 (mean VS:S concentration ratio = 1.98) in very severe patients presenting with characteristic respiratory failure compared to severe patients (Figure 2A). Another 3 immune mediators––GM-CSF, MIP-1β, and IL-2––were significantly enhanced in plasma derived from all severe patients with characteristic neurological symptoms compared to both mild patients and healthy controls (Figure 2B); this result suggested that these specific mediators maybe relevant to neurological damage. We also found a marked increase in IL-23 (mean S:M concentration ratio = 6.18) and IL-33 (mean S:M concentration ratio = 3.20) in severe patients presenting with neurological manifestations compared to mild patients (Figure 2B), although significance was not attained due to wide variability among individual patients (IL-23 levels ranged from 46.42–36615.08 pg/mL in severe patients, and IL-33 levels ranged from 6.75–987.71 pg/mL in severe patients). Finally, another 5 factors––IFNα2a, MIP-1α, IP-10, IL-6, and IL-8––were also significantly elevated in all EV71–infected HFMD patients as compared to healthy controls; however, no significant differences between individual groups within the EV71–infected patients were observed (Figure 2C).

**Figure 3:**
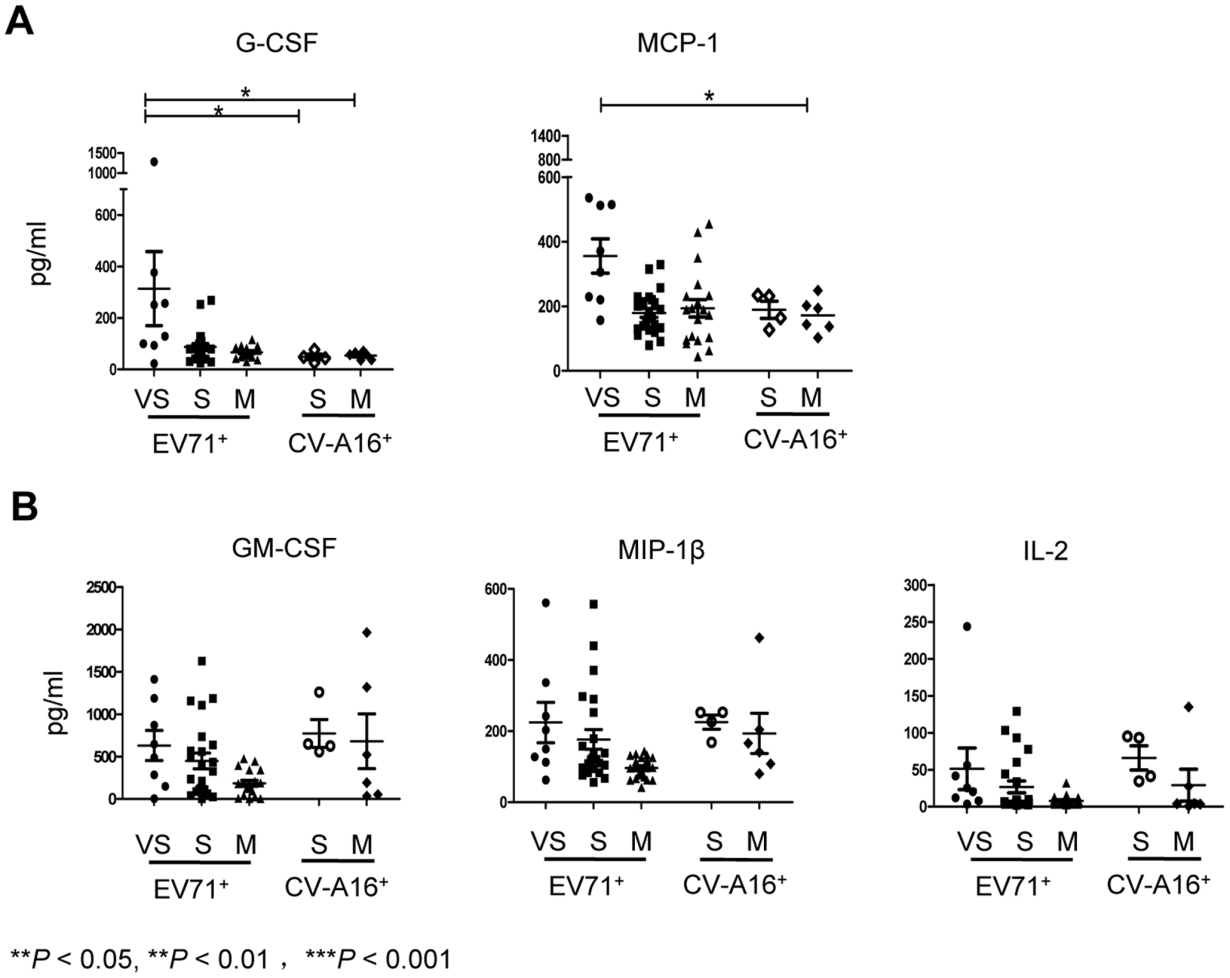
Plasma cytokine/chemokine expression pattern was compared between EV71– and CA16–positive patients. A panel of 12 immune mediators was evaluated in plasma samples from CA16–positive patients with neurological manifestations: S = 4; M = 6. (A) The expression pattern of G-CSF and MCP-1 was consistent between EV71– and CA16–mediated mild and severe HFMD patients, and G-CSF and MCP-1 were significantly higher in EV71–mediated very severe HFMD patients with respiratory failure as compared to all other groups. (B) The expression pattern of GM-CSF, IL-2, and MIP-1β were consistent in both EV71– and CA16–positive mild and severe patients. The unpaired Student’s *t*-test and non-parametric ANOVA test was used to compare variables between the indicated 2 groups. *P<0.05, **P<0.01,***P<0.001. Data are representative of at least 2 experiments.

CA16 is believed to be the second major causative pathogen inducing HFMD [9]. Using the same symptom classification for HFMD regardless of viral etiology, we tested whether similar immune mediators were elicited upon the onset of similar symptoms by comparing cytokine/chemokine levels between EV71– and CA16–positive HFMD patients. No patient in CA16 infection group suffered quickly developed acute respiratory failure in this study. Interestingly, severe and mild HFMD patients induced by either CA16 or EV71 exhibited similar expression patterns and levels in plasma. Importantly, G-CSF and MCP-1 expression in very severe EV71–positive patients were significantly higher than in either EV71– or CA16–positive mild patients (Figure 3A), implying these 2 factors may act as potential predictors of severe neurological damage with acute respiratory failure of EV71 infected HFMD patients. Moreover, GM-CSF, MIP-1β, and IL-2 expression exerted similarly elevated levels in both EV71– and CA-16–infected severe patients with uncomplicated neurological presentations as compared to mild patients (Figure 3B).

### Five Immune Mediators are Elevated in CSF as Compared to Plasma in Patients with Neurological Complications

**Figure 4:**
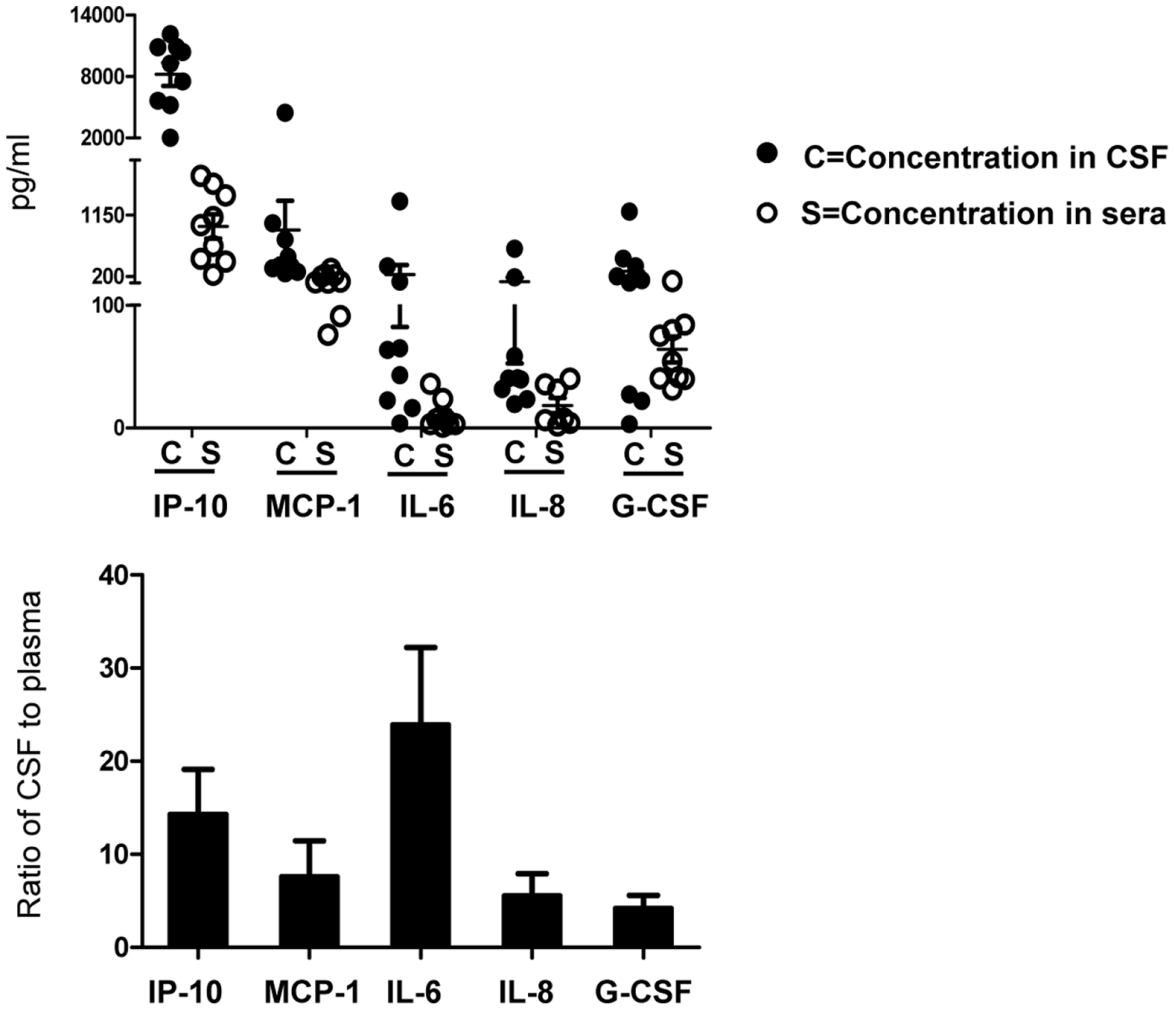
Five immune mediators are elevated in CSF as compared to plasma in EV71-positive patients with neurological complications. Cytokine/chemokine expression was evaluated in CSF samples from 9 severe patients with neurological manifestations. Five cytokines/chemokines levels were significantly higher in CSF as compared to plasma. Absolute cytokine/chemokine concentration in plasma (open circles) and CSF (solid circles) (upper panel); ratio of CSF:plasma concentrations >1 (lower panel). Data are expressed as mean ± SEM, and are representative of at least 2 experiments.

To determine whether immune mediators are locally secreted at the site of neurological damage, we examined the above-mentioned cytokines/chemokines in CSF samples from severe patients with neurological manifestations and compared the cytokine/chemokine levels between CSF and plasma collected at the same time point from each patient. The quantified protein levels of IL-8, IP-10, and MCP-1 chemokines as well as the pleiotropic cytokines IL-6 and G-CSF from individual patients were higher in CSF as compared to plasma (Figure 4, upper panel), with the following mean CSF:plasma concentration ratios: IL-8 (5.54±2.39), IP-10 (14.29±4.82), MCP-1 (7.58±3.86), IL-6 (23.91±8.31), and G-CSF (4.18±1.42) (Figure 4, lower panel). These data indicate that the 5 factors described here expressed higher level in CSF and may be useful in monitoring and predicting progression to neurological damage by comparing dynamics changes of their expression level. In contrast to these 5 factors, the other surveyed immune factors were mainly found in plasma, as only minute quantities of these factors were detected in CSF samples (Figure S2, upper panel). Indeed, the mean plasma:CSF concentration ratios for these cytokines reached as high as 141.12 (Figure S2, lower panel).

### EV71 VP-1–specific Antibodies are Elevated during Disease Development, and the CD19^+^HLADR^+^ Mature B cell Subset Frequency in Peripheral Blood is Temporally Increased

**Figure 5:**
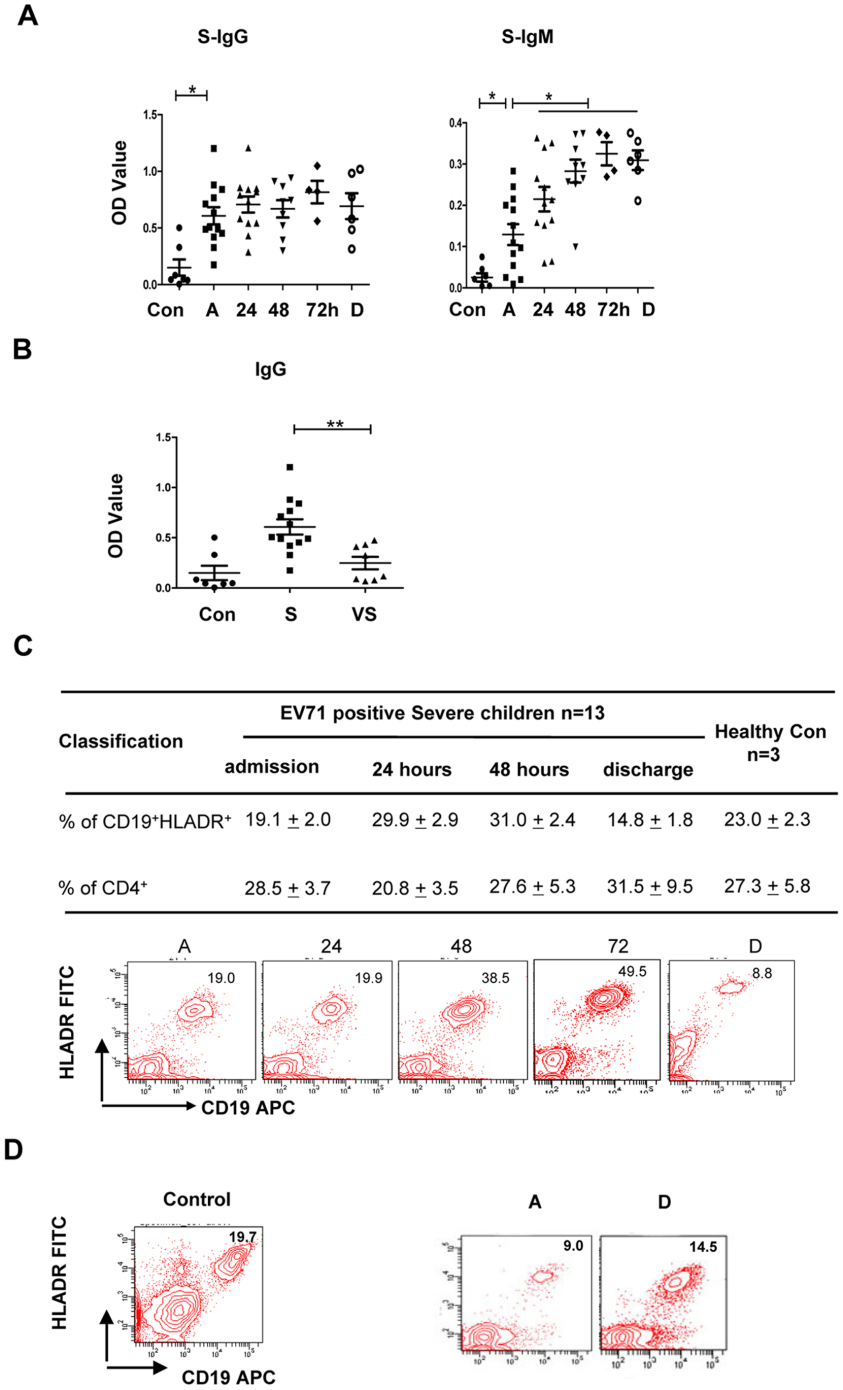
Production of EV71 VP-1–specific IgG and IgM antibodies in patients with increased frequency of CD19^+^HLA-DR^+^ cells. (A) IgG and IgM antibodies were rapidly increased and were markedly higher in plasma from severe patients (n = 13) compared with normal controls (n = 7). The differences between two time points of every individuals were determined by Wilcoxon’s Sign Rank Test. *P<0.05. (B) Very severe patients with acute respiratory failure (n = 8) have lower IgG secretion as compared to patients with uncomplicated neurological manifestations (n = 13), which were both examined before treatment. The unpaired Student’s *t*-test and non-parametric ANOVA test were used to compare variables between the indicated 2 groups. **P<0.01. (C) Frequency of CD19^+^HLA-DR^+^ lymphocytes in peripheral blood of severe patients (n = 13) and healthy controls (n = 3). Data are expressed as the percentage of the examined cell subset within gated lymphocytes. The lower panel showing representative data from a severe patient indicated that CD19^+^HLADR^+^ frequency increased during the course of hospitalization. (D) Representative FACS data examining CD19^+^ cells from a healthy control and a mild patient with no neurological manifestations.

Although cellular immune response has been confirmed an important role in the disease of HFMD, some of the immune mediators identified here are also important for an effective B cell-mediated humoral response, including neutralizing antibody production [18], [19]. We examined EV71 VP-1–specific IgG and IgM antibody levels in patient plasma. VP-1–antigen specific antibodies were quickly and significantly enhanced in severe patients, with sustained IgG expression and gradually increasing IgM secretion compared to healthy controls (Figure 5A). Interestingly, the levels of IgG (Figure 5B), but not IgM (data not shown), were observed to be significantly lower in the very severe patient group with acute respiratory failure compared to the severe group before treatment, suggesting the titer of VP-1 specific antibodies may be related to the disease. Our data are consistent with results from another study reporting no differences in IgM titers among patient groups with differing severity [13], although data regarding IgG titers were not included. In our study, both IgG and IgM antibody levels were similar between the severe and mild patient groups, revealing that EV71 viral infection may induce VP-1–specific antibody production soon after infection (Figure S3).

To further evaluate B cell involvement in EV71–mediated HFMD, we measured the frequency of CD19^+^HLA-DR^+^ cells, which represent a mature B cell subset phenotype, by FACS analysis. Strikingly, the frequency of CD19^+^HLA-DR^+^ lymphocytes increased during patient hospitalization and peaked at 48–72 h after glucocorticoid treatment (Figure 5C). Results from one representative patient showed a 2-fold increase in CD19^+^HLA-DR^+^ cell frequency 72 h after hospitalization as compared to admission (from 19.0% to 49.5%), which then returned to admission levels at discharge (Figure 5C). We also analyzed CD19^+^ B cell frequency in mild cases and observed a slight increase at discharge (14.5%) as compared to admission (9.0%) (Figure 5D, right). This frequency at discharge was slightly lower than the peripheral CD19^+^ frequency range found in healthy controls, which varied from 19.7–27.3%, as shown in one representative healthy control in Figure 5D (left).

## Discussion

Infection with EV71 leads to a rapidly progressing, life-threatening, and severe neurological disease in young children and is the major cause of fatal HFMD outbreaks within the Asia Pacific Region [13]–[15], [20], [21]. However, the mechanism by which host immunity is activated by and staves off this viral infection remains unknown. Increasing evidence suggests that both pro- and anti-inflammatory cytokines may play a central role in EV71–infection-mediated HFMD that presents with neurological damage and pulmonary/cardiac failure. Herein, we comprehensively screened a large panel of 120 immune factors in HFMD patients as compared to age-matched healthy controls. We further confirmed these results in a selection of 30 cytokines and chemokines in samples derived from larger pool of EV71– and CA16–positive HFMD patients as compared to age-matched healthy controls; additionally, we analyzed these selected immune mediators in patient groups stratified by symptom severity. Our results showed that (1) GM-CSF, MIP-1β, IL-2, IL-33, and IL-23 secretion was elevated in patients who rapidly developed disease and presented with uncomplicated neurological damage; and (2) G-CSF and MCP-1 were distinguishably secreted in EV71 infected very severe patients presenting with acute respiratory failure. Previous studies using other methods also described that one or more of the following cytokines/chemokines were elevated in the blood of severe HFMD patients: anti-inflammatory IL-10; pro-inflammatory IL-1β, IL-2, IL-13, and IFN-γ; pleiotropic IL-6; and IP-10 and MCP-1 chemokines [1], [2], [7]. While these previous studies provide fundamental and useful information on pathogen-mediated inflammation, the field currently lacks a more comprehensive and detailed comparison of immune mediators in patients with different clinical degrees of severity that are studied in parallel and within a single study.

We thus addressed this concern in our present study. Some of the expression patterns identified in the present study come from cytokines or chemokines that, to our knowledge, have not been previously examined in patients with differing clinical outcomes in parallel and within a single study. Our data offer several important implications in understanding the immune response during EV71–mediated HFMD, as follows: (a) GM-CSF, IL-2, and MIP-1β were significantly enhanced in the plasma from very severe and severe patients presenting with neurological damage as compared to mild patients and age-matched healthy controls (Figure 2B), suggesting that these factors may potentially predict damage to the nervous system induced by EV71 infection. (b) IL-23 and IL-33 were also markedly increased, especially in severe patients, with large variability exhibited within some patient groups (Table S1). Several studies demonstrate that IL-23 plays a critical role against viral and bacterial infection and in generating airway inflammation [22]–[24]. Moreover, IL-23 stimulates IFN-γ production and enhances cell-mediated immune responses, including CTLs [25]–[27]. IL-33 is a tissue-derived cytokine that drives Th2 cell polarization, is involved in lung-related diseases [28], [29], is released in damaged tissues or necrotic cells, and acts as an alarmin [30], [31] in host defense against pathogens. Our data may therefore have broader implications for pathologies associated with Th1, Th2, and other pleiotropic cytokine profile changes away from homeostasis, where impaired production may correlate with worsening disease. (c) IFNα2a, IL-6, IL-8, MIP-1α, and IP-10 were additionally elevated in both severe and mild patients (Figure 2C) compared to healthy controls. While this result confirms the involvement of these cytokines found in some of the earlier research, further investigation is needed to determine how each of these may be relevant to EV71 infection and disease severity. (d) Among the surveyed immune mediators, G-CSF and MCP-1 were significantly enhanced in the very severe patient group, which contained patients presenting with respiratory failure, as compared to the other patient groups and to healthy controls (Figure 2A). Since G-CSF is produced by a wide range of cells in response to certain stimuli [32] and promotes cell differentiation and proliferation [33], further study into whether elevated G-CSF levels exaggerate the already imbalanced cell activation that is partially responsible for HFMD pathogenesis is warranted. We also performed kinetic analysis of cytokine/chemokine expression at the above-mentioned time points during patient hospitalization. Figure S1 showed that GM-CSF, MIP-1β, IL-2, and IL-33 all similarly presented stable protein levels in plasma that initially increase, peak at 48–72 hours after hospitalization, and then decrease again at discharge. In contrast, MCP-1 expression gradually increased over time, and IL-23 levels sharply decreased almost to baseline level at discharge. These data suggest that the elevated immune mediator levels present in patients before treatment may decrease at a slower rate than the reduction of the physical symptoms they experience. (e) For patients with neurological manifestations, G-CSF, IL-8, MCP-1, IP-10, and IL-6 levels were remarkably higher in CSF than in plasma, suggesting that they may be predominant mediators induced when neurological damage occurs in the CSF. Unfortunately, because obtaining sufficient numbers of eligible CSF samples from healthy controls is difficult, we could not compare the levels or CSF:plasma ratios of these factors between HFMD patients and controls. Previous evidence suggests that MCP-1 levels may be higher in CSF (up to 683 pg/mL) than in serum of healthy adults [34], which may argue against being able to use this factor for diagnostic purpose unless a clear difference in the magnitude between the CSF and serum in HFMD patients as compared to healthy age-matched controls can be demonstrated. However, our data are consistent with previous findings that IP-10, MCP-1, and IL-8 levels in CSF are elevated above plasma levels in patients presenting with CNS damage [35]. Because chemokines are produced by CNS cells within hours after injury and are thought to mediate recruitment and activation of mononuclear phagocytes [36], [37], we suppose that increased chemokine levels in the CSF of HFMD patients may mediate early regulation and recruitment of protective immunity that reduce severe tissue damage. To address whether the cytokine/chemokine expression pattern correlates in any way to viral load in the patients, we also performed Real-time quantitative RT-PCR to examine virus load from throat swabs. Our results showed that low copies of viral load (copies in samples from very severe group were: 10601, 1652, 1052, 911, 498, 412; copies in samples from severe group were: 1185, 518, 425, 305, 166, 148; copies in samples from mild group were: 1358, 818, 803, 621, 348, Figure S4) was found in examined samples and did not exhibit any particular correlation with cytokine/chemokine expression level. Although one sample (from very severe group) had high copies of 10601, its corresponding cytokine expression pattern was similar as samples from other very severe patients. The substantially increased concentrations of proinflammatory serum cytokines and chemokines expressed in patient were compatible with clinical severity of the disease (Figure 1). Since the cause of cytokine/chemokine release during EV71 infection is still unclear, further investigation is needed to explore the sources for each of these cytokines and chemokines as well as the underlying mechanisms for how their presence is associated with infection and inflammation in this disease and is regulated. Answering these unknowns may help to translate these findings from bench to bedside.

We also found in our study that the frequency of CD19^+^HLA-DR^+^ mature B cells was temporally enriched in the periphery soon after symptomatic therapy and glucocorticoid treatment in HFMD patients. Fully activated B cells play an important role in Th1 differentiation via B cell-derived IL-12 and for amplifying and maintaining a T cell response [38]. Whether and when systemic glucocorticoids should be used to treat high fever, like that experienced by severe and very severe HFMD patients, is still controversial; when we examined 3 severe HFMD patients at the height of high fever in our study population that were not treated with glucocorticoid treatment, no significant or even slight increase in CD19^+^HLA-DR^+^ cells was found (data not shown). Further studies with a wider range of patient samples are required to determine if similar results can be obtained and which factors influence the change in mature B cell frequency during disease. If so, this may lead to more discussion in the field regarding the optimal use of glucocorticoid treatment to control disease but not to impair protective immunity against pathogens. Taken together, our results contribute to a comprehensive understanding of EV71 infection-induced cytokine and chemokine production, which may have therapeutic and/or prognostic value in managing severe hand, foot, and mouth disease patients with neurological damage.

## Supporting Information

Figure S1 **Kinetics of cytokine/chemokine response in EV71-positive severe patients (n = 9).** Blood samples from 9 severe patients were successfully collected at 4 time points, including admission, 24 h, 48–72 h, and discharge. Luminex-based cytokine bead array was performed to examine dynamic cytokine/chemokine expression at each time point. Data are presented as mean ± SEM. Wilcoxon’s Sign Rank Test was used to compare the differences between every two time points of individuals. *P<0.05, **P<0.01.
(TIF)

Figure S2 **Some immune mediators are more highly expressed in plasma as compared to CSF in EV71-positive patients with neurological complications.** Cytokine/chemokine expression was evaluated in CSF samples from 9 severe patients with neurological manifestations. Nine factors are predominantly expressed in plasma. Absolute cytokine/chemokine concentration in plasma (open circles) and CSF (solid circles) (upper panel); Ratio of plasma:CSF concentrations >1 (lower panel). Data are expressed as mean ± SEM, and are representative of at least 2 experiments.
(TIF)

Figure S3 **Comparison of IgG and IgM antibody levels in EV71-positive severe (n = 13) and mild patients (n = 13) at time points before treatment.** The unpaired Student’s *t*-test and non-parametric ANOVA test were used to compare variables between the indicated 2 groups. *P<0.05, **P<0.01,***P<0.001.
(TIF)

Figure S4 **Virus load determination in EV71-positive patients.** The copies of virus were determined in EV71 positive patients according to the standard curve (A). The dissociation curve (B) presented the specificity of amplification.
(TIF)

Table S1 **Seventeen cytokine/chemokines were analyzed in patients and controls.**
(PPTX)
