# Correction: Comparative Study of the Cytokine/Chemokine Response in Children with Differing Disease Severity in Enterovirus 71-Induced Hand, Foot, and Mouth Disease

**DOI:** 10.1371/annotation/f582a7ef-3b3e-4e95-9522-23f0f3f77cde

**Published:** 2013-07-08

**Authors:** Yan Zhang, Haiying Liu, Linghang Wang, Fan Yang, Yongfeng Hu, Xianwen Ren, Guojun Li, Yang Yu, Shaoxia Sun, Yufen Li, Xinchun Chen, Xingwang Li, Qi Jin

The name of the eighth author was given incorrectly. The correct name is: Yu Yang. 

