# Supplementary figures and images for: Comparative Study of the Cytokine/Chemokine Response in Children with Differing Disease Severity in Enterovirus 71-Induced Hand, Foot, and Mouth Disease
Source: PLoS One. 2013 Jun 28;8(6):e67430. doi: 10.1371/journal.pone.0067430 (PMC3696071; doi:10.1371/journal.pone.0067430)

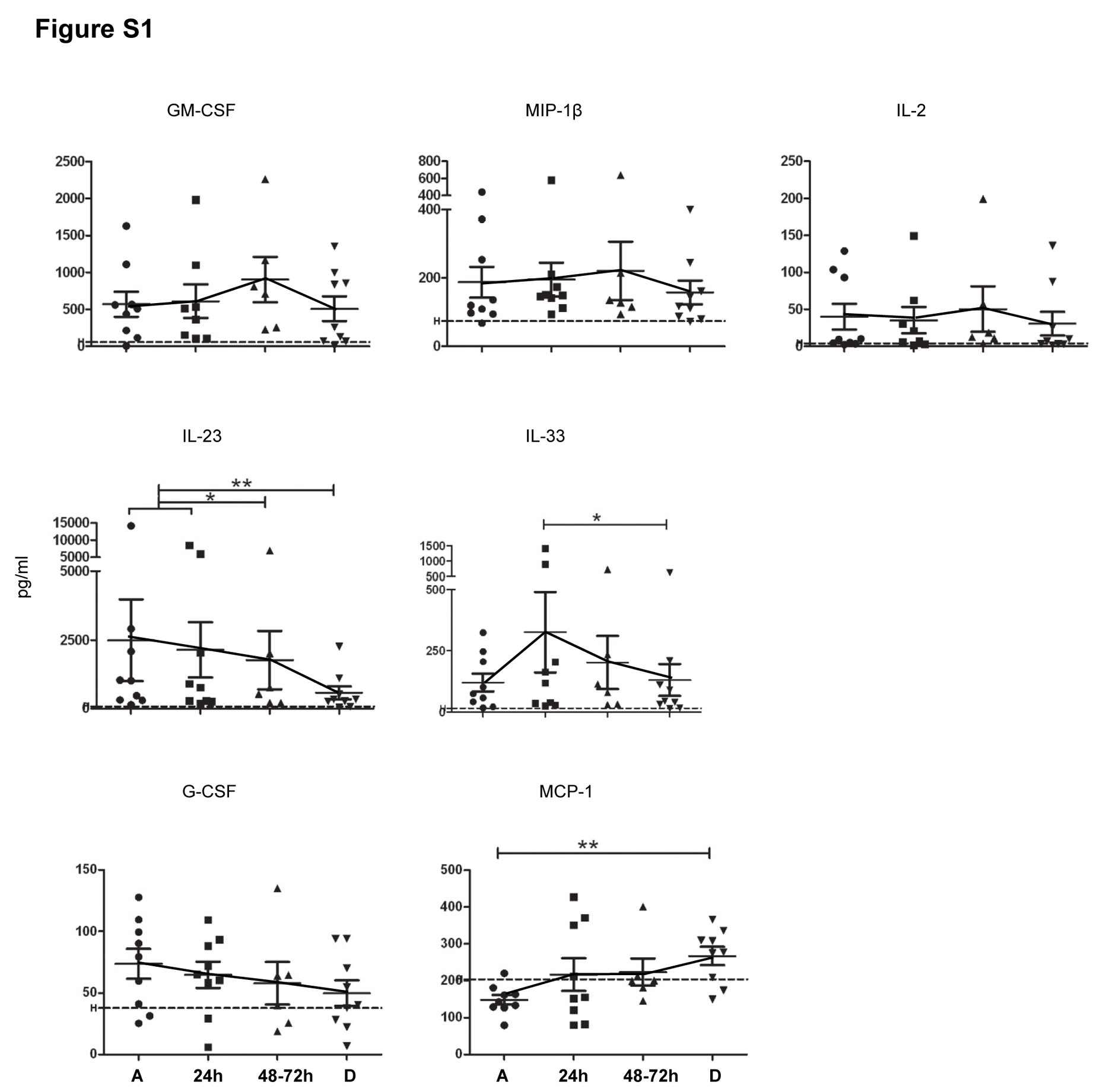

Supplement: Figure S1 — Kinetics of cytokine/chemokine response in EV71-positive severe patients (n = 9). Blood samples from 9 severe patients were successfully collected at 4 time points, including admission, 24 h, 48–72 h, and discharge. Luminex-based cytokine bead array was performed to examine dynamic cytokine/chemokine expression at each time point. Data are presented as mean ± SEM. Wilcoxon’s Sign Rank Test was used to compare the differences between every two time points of individuals. *P<0.05, **P<0.01. (TIF) [file pone.0067430.s001.tif]

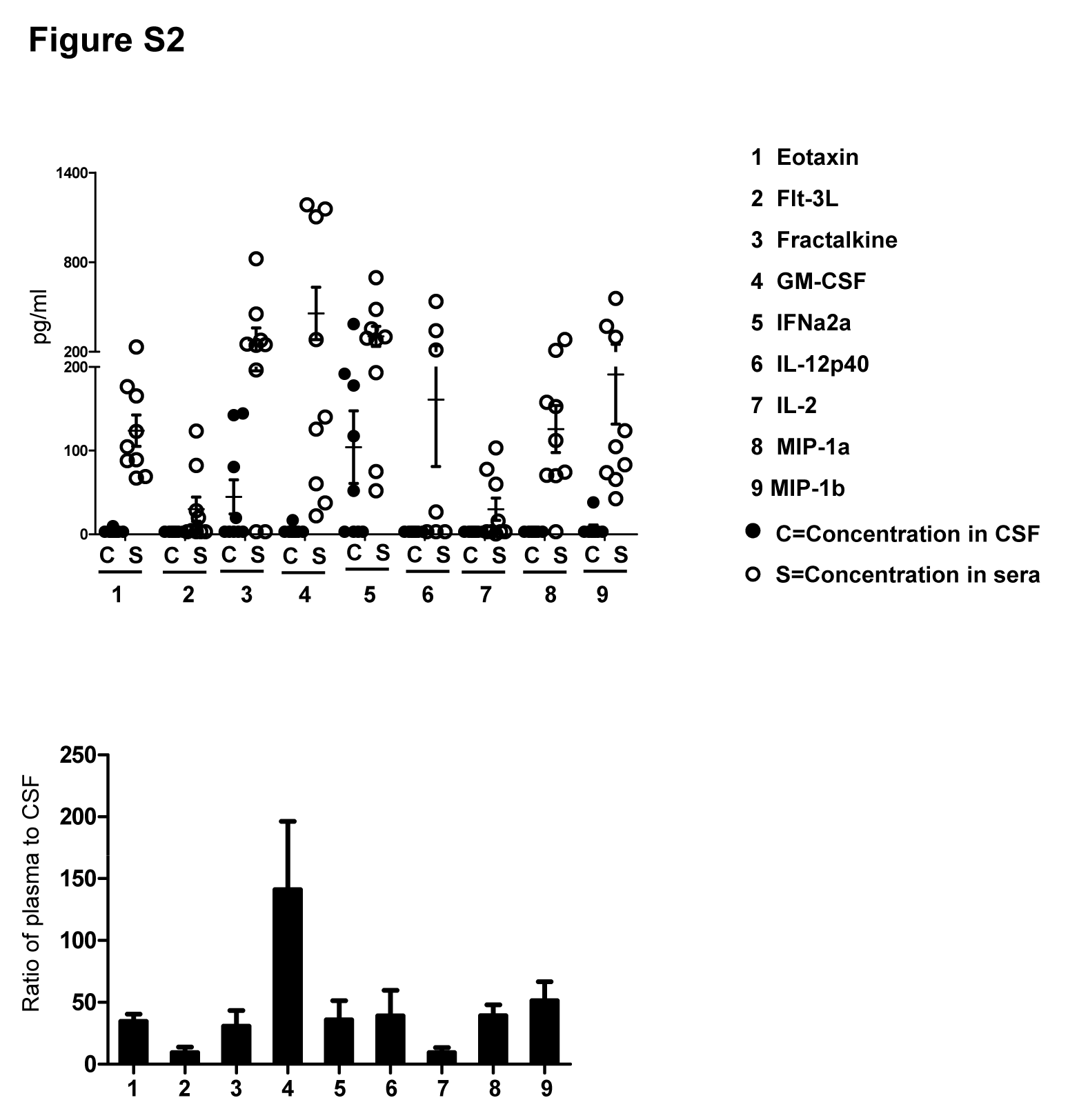

Supplement: Figure S2 — Some immune mediators are more highly expressed in plasma as compared to CSF in EV71-positive patients with neurological complications. Cytokine/chemokine expression was evaluated in CSF samples from 9 severe patients with neurological manifestations. Nine factors are predominantly expressed in plasma. Absolute cytokine/chemokine concentration in plasma (open circles) and CSF (solid circles) (upper panel); Ratio of plasma:CSF concentrations >1 (lower panel). Data are expressed as mean ± SEM, and are representative of at least 2 experiments. (TIF) [file pone.0067430.s002.tif]

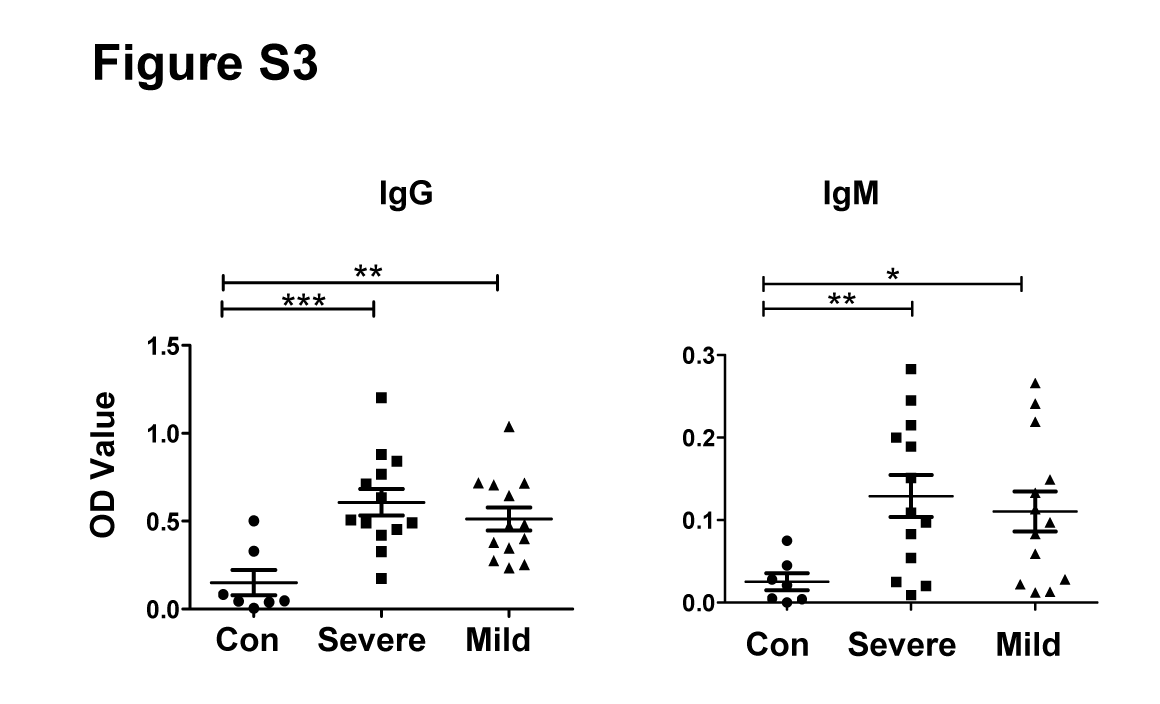

Supplement: Figure S3 — Comparison of IgG and IgM antibody levels in EV71-positive severe (n = 13) and mild patients (n = 13) at time points before treatment. The unpaired Student’s t-test and non-parametric ANOVA test were used to compare variables between the indicated 2 groups. *P<0.05, **P<0.01,***P<0.001. (TIF) [file pone.0067430.s003.tif]

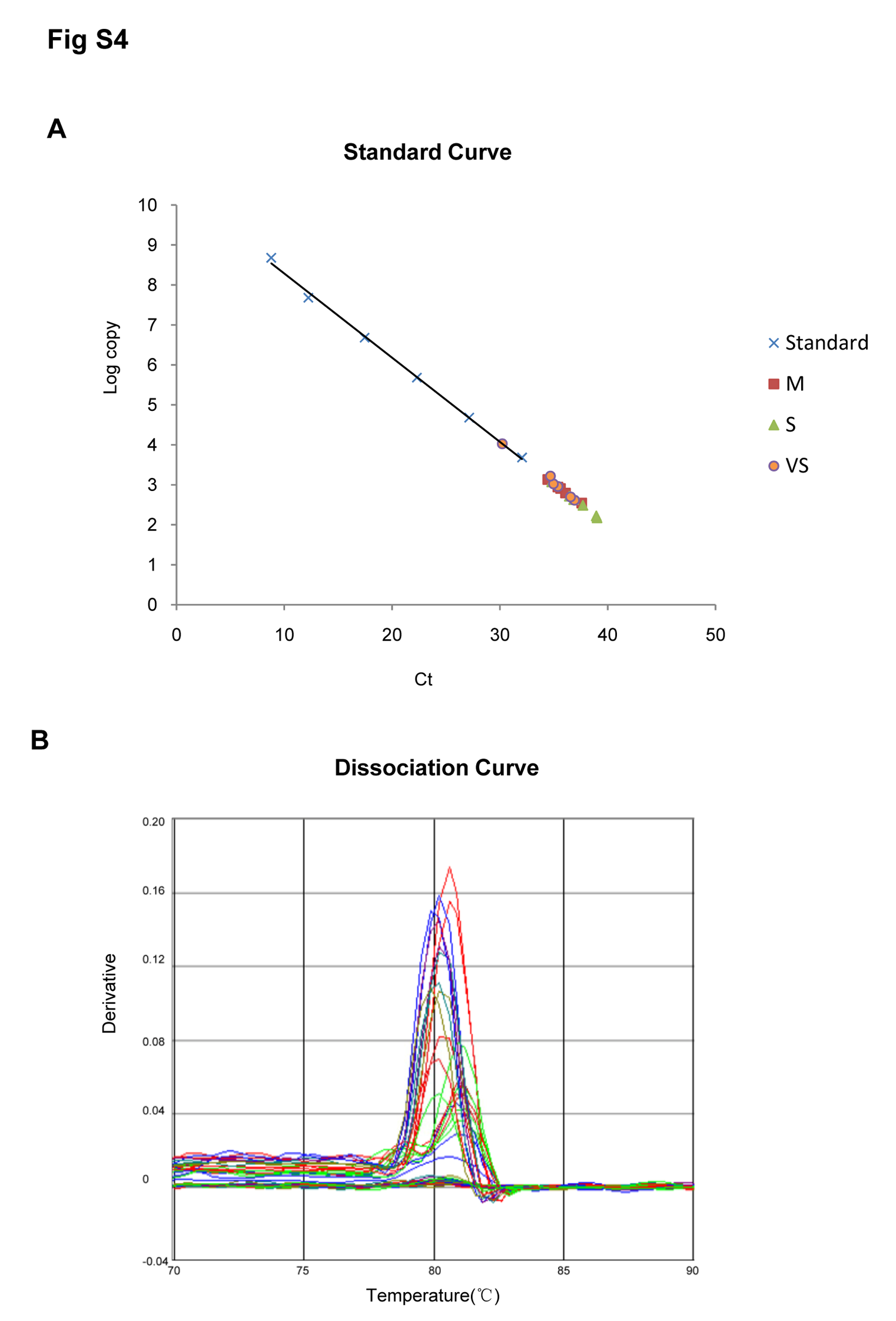

Supplement: Figure S4 — Virus load determination in EV71-positive patients. The copies of virus were determined in EV71 positive patients according to the standard curve (A). The dissociation curve (B) presented the specificity of amplification. (TIF) [file pone.0067430.s004.tif]
