# Supplementary material for: Comparative Study of the Cytokine/Chemokine Response in Children with Differing Disease Severity in Enterovirus 71-Induced Hand, Foot, and Mouth Disease
Source: PLoS One. 2013 Jun 28;8(6):e67430. doi: 10.1371/journal.pone.0067430 (PMC3696071; doi:10.1371/journal.pone.0067430)
Supplement: Table S1 — Seventeen cytokine/chemokines were analyzed in patients and controls. (PPTX) [file pone.0067430.s005.pptx]

## Slide 1
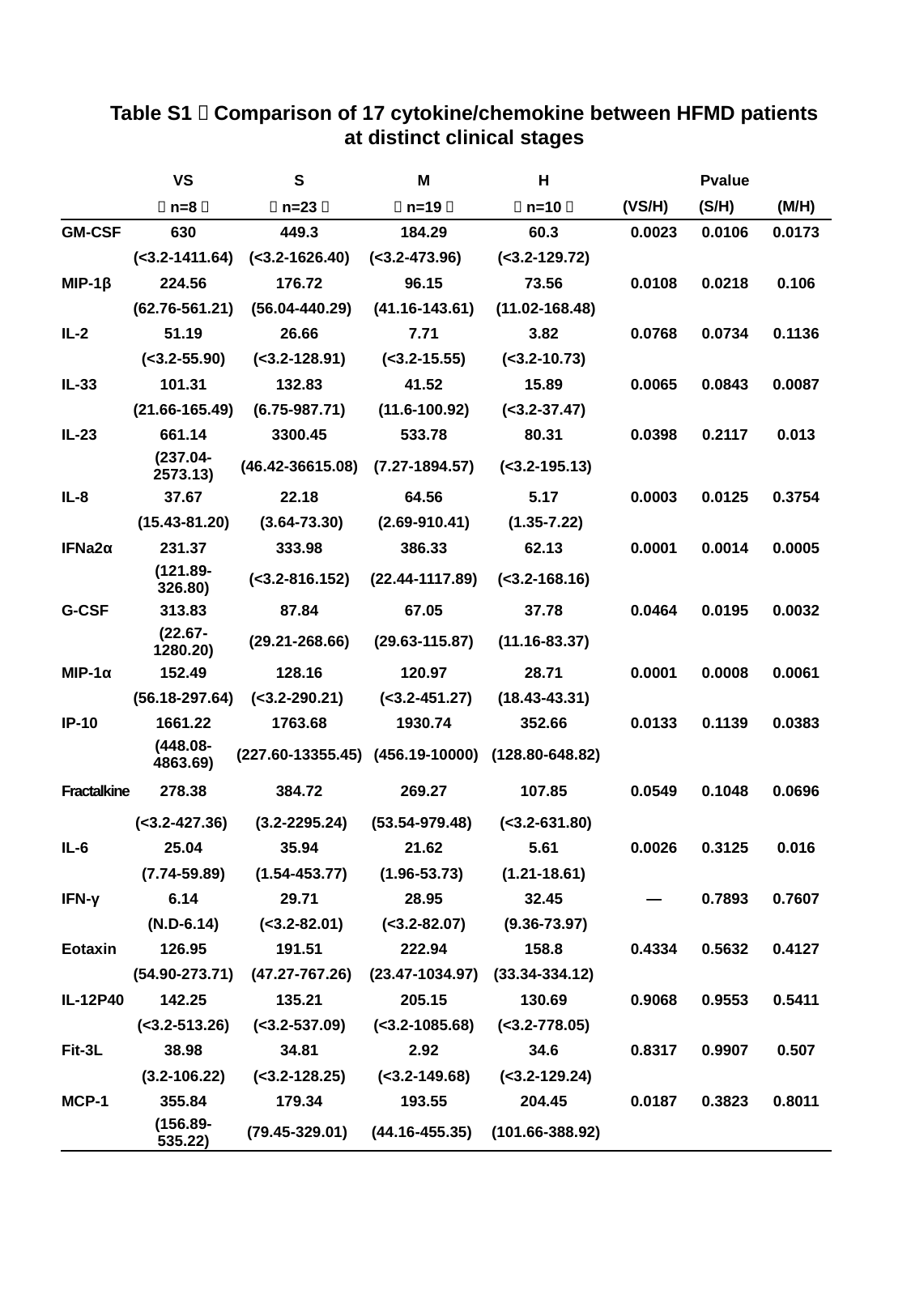

Table S1：Comparison of 17 cytokine/chemokine between HFMD patients at distinct clinical stages
| | VS | S | M | H | | Pvalue | | |
| --- | --- | --- | --- | --- | --- | --- | --- | --- |
| | （n=8） | （n=23） | （n=19） | （n=10） | | (VS/H) | (S/H) | (M/H) |
| GM-CSF | 630 | 449.3 | 184.29 | 60.3 | | 0.0023 | 0.0106 | 0.0173 |
| | (<3.2-1411.64) | (<3.2-1626.40) | (<3.2-473.96) | (<3.2-129.72) | | | | |
| MIP-1β | 224.56 | 176.72 | 96.15 | 73.56 | | 0.0108 | 0.0218 | 0.106 |
| | (62.76-561.21) | (56.04-440.29) | (41.16-143.61) | (11.02-168.48) | | | | |
| IL-2 | 51.19 | 26.66 | 7.71 | 3.82 | | 0.0768 | 0.0734 | 0.1136 |
| | (<3.2-55.90) | (<3.2-128.91) | (<3.2-15.55) | (<3.2-10.73) | | | | |
| IL-33 | 101.31 | 132.83 | 41.52 | 15.89 | | 0.0065 | 0.0843 | 0.0087 |
| | (21.66-165.49) | (6.75-987.71) | (11.6-100.92) | (<3.2-37.47) | | | | |
| IL-23 | 661.14 | 3300.45 | 533.78 | 80.31 | | 0.0398 | 0.2117 | 0.013 |
| | (237.04-2573.13) | (46.42-36615.08) | (7.27-1894.57) | (<3.2-195.13) | | | | |
| IL-8 | 37.67 | 22.18 | 64.56 | 5.17 | | 0.0003 | 0.0125 | 0.3754 |
| | (15.43-81.20) | (3.64-73.30) | (2.69-910.41) | (1.35-7.22) | | | | |
| IFNa2α | 231.37 | 333.98 | 386.33 | 62.13 | | 0.0001 | 0.0014 | 0.0005 |
| | (121.89-326.80) | (<3.2-816.152) | (22.44-1117.89) | (<3.2-168.16) | | | | |
| G-CSF | 313.83 | 87.84 | 67.05 | 37.78 | | 0.0464 | 0.0195 | 0.0032 |
| | (22.67-1280.20) | (29.21-268.66) | (29.63-115.87) | (11.16-83.37) | | | | |
| MIP-1α | 152.49 | 128.16 | 120.97 | 28.71 | | 0.0001 | 0.0008 | 0.0061 |
| | (56.18-297.64) | (<3.2-290.21) | (<3.2-451.27) | (18.43-43.31) | | | | |
| IP-10 | 1661.22 | 1763.68 | 1930.74 | 352.66 | | 0.0133 | 0.1139 | 0.0383 |
| | (448.08-4863.69) | (227.60-13355.45) | (456.19-10000) | (128.80-648.82) | | | | |
| Fractalkine | 278.38 | 384.72 | 269.27 | 107.85 | | 0.0549 | 0.1048 | 0.0696 |
| | (<3.2-427.36) | (3.2-2295.24) | (53.54-979.48) | (<3.2-631.80) | | | | |
| IL-6 | 25.04 | 35.94 | 21.62 | 5.61 | | 0.0026 | 0.3125 | 0.016 |
| | (7.74-59.89) | (1.54-453.77) | (1.96-53.73) | (1.21-18.61) | | | | |
| IFN-γ | 6.14 | 29.71 | 28.95 | 32.45 | | — | 0.7893 | 0.7607 |
| | (N.D-6.14) | (<3.2-82.01) | (<3.2-82.07) | (9.36-73.97) | | | | |
| Eotaxin | 126.95 | 191.51 | 222.94 | 158.8 | | 0.4334 | 0.5632 | 0.4127 |
| | (54.90-273.71) | (47.27-767.26) | (23.47-1034.97) | (33.34-334.12) | | | | |
| IL-12P40 | 142.25 | 135.21 | 205.15 | 130.69 | | 0.9068 | 0.9553 | 0.5411 |
| | (<3.2-513.26) | (<3.2-537.09) | (<3.2-1085.68) | (<3.2-778.05) | | | | |
| Fit-3L | 38.98 | 34.81 | 2.92 | 34.6 | | 0.8317 | 0.9907 | 0.507 |
| | (3.2-106.22) | (<3.2-128.25) | (<3.2-149.68) | (<3.2-129.24) | | | | |
| MCP-1 | 355.84 | 179.34 | 193.55 | 204.45 | | 0.0187 | 0.3823 | 0.8011 |
| | (156.89-535.22) | (79.45-329.01) | (44.16-455.35) | (101.66-388.92) | | | | |
